# PARP inhibitors and breast cancer: from therapeutic breakthrough to resistance challenge

**DOI:** 10.1038/s12276-026-01673-8

**Published:** 2026-04-10

**Authors:** Weiyun Wang, Chenghui Cai, Sisi Qin, Liujun He, Qinhao Liang, Siyao Tang, Jie Xie, Ting Long, Jing Hou, Wootae Kim, Fei Zhao

**Affiliations:** 1 Guizhou University of Traditional Chinese Medicine, Guiyang, China; 2 Hunan Research Center of the Basic Discipline for Cell Signaling, College of Biology, Hunan University, Changsha, China; 3 Department of Pathology, University of Chicago, Chicago, IL USA; 4 Department of Breast Surgery, Guizhou Provincial People’s Hospital, Guiyang, China; 5 Zunyi Medical University, Zunyi, China; 6 Guizhou University Medical College, Guiyang, China; 7 Guizhou Medical University, Guiyang, China; 8 Department of Integrated Biomedical Science, Soonchunhyang Institute of Medi-bio Science, Soonchunhyang University, Cheonan, Republic of Korea; 9 Institute for Molecular Metabolism Innovation, Soonchunhyang University, Asan, Republic of Korea

**Keywords:** Cancer therapeutic resistance, Prognostic markers, Prognostic markers, Breast cancer

## Abstract

Breast cancer remains the leading cause of cancer-related mortality among women worldwide. Poly(ADP-ribose) polymerase (PARP) inhibitors have emerged as a critical therapeutic option, particularly for patients with triple-negative breast cancer and other HER2-negative metastatic breast cancer harboring *BRCA* mutations. Despite their clinical success, the emergence of primary and acquired resistance to PARP inhibitors poses a significant challenge, limiting their long-term effectiveness. Here we provide a comprehensive overview of the mechanisms underlying the action of PARP inhibitors, as well as their clinical development and application. In addition, we discuss the factors driving resistance and potential strategies to overcome it in the context of PARP inhibitors.

## Overcoming resistance to PARP inhibitors in breast cancer

Breast cancer is a leading cause of cancer-related deaths among women globally. The study highlights the increasing burden of breast cancer, especially in low-resource areas, and the need for personalized treatment strategies. Researchers have focused on classifying breast cancer into subtypes to improve treatment precision. This article reviews the role of poly(ADP-ribose) polymerase (PARP) inhibitors, a type of targeted therapy approved by the US Food and Drug Administration in 2018 for certain breast cancers with *BRCA* mutations. PARP inhibitors work by blocking enzymes involved in DNA repair, making them effective against cancer cells with specific genetic defects. The study discusses how PARP inhibitors have shown promise in clinical trials but face challenges such as resistance, where cancer cells adapt and become less responsive to treatment. Researchers are exploring ways to overcome this resistance, such as combining PARP inhibitors with other therapies.

This summary was initially drafted using artificial intelligence, then revised and fact-checked by the author.

## Introduction

Breast cancer (BC) is among the primary causes of cancer-related mortality among women worldwide. A 2025 analysis, utilizing global data from the year of 2022, confirms that BC remains the leading cause of cancer incidence and a major cause of mortality in women, responsible for 25% of new cases and 15.5% of deaths. Projections indicate a worsening burden, with cases and deaths expected to rise by 38% and 68%, respectively, by the year 2050, disproportionately impacting low-resource settings^1^.

BC exhibits considerable heterogeneity, with distinct subtypes demonstrating diverse characteristics that require personalized therapeutic strategies. To facilitate precise treatment decisions, BC is classified into various molecular subtypes. The most recognized classification system categorizes BC into three subtypes on the basis of the expression of estrogen receptor (ER), progesterone receptor (PR) and human epidermal growth factor receptor 2 (HER2): ER-positive, HER2-positive, and triple-negative BC (TNBC). Advances in treatment strategies, including surgery, radiotherapy, chemotherapy, endocrine therapy and targeted therapy, have substantially improved outcomes for patients across different subtypes. Among these, targeted therapy is particularly notable for its ability to selectively bind to specific molecular targets in tumors, thereby enhancing both the precision and safety of treatment.

Poly(ADP-ribose) polymerase (PARP) inhibitors were approved by the US Food and Drug Administration (FDA) in 2018 as targeted therapeutics for BC, primarily indicated for patients with HER2-negative metastatic BC (mBC) who harbor *BRCA* mutations^2^. The clinical potential of PARP inhibitors (PARPi) was demonstrated in the OlympiA trial, which demonstrated that PARPi meaningfully improved invasive disease-free survival (iDFS) in patients with early-stage, high-risk BC^3^. Furthermore, the OlympiAD and EMBRACA trials confirmed that PARPi prolonged clinical progression-free survival (PFS) benefits in patients with advanced *BRCA*-mutated BC^4–6^.

The long-term efficacy of PARPi in *BRCA*-mutated BC is frequently limited by acquired resistance. A key mechanism is the restoration of BRCA function through secondary somatic mutations. This form of PARPi resistance is context dependent and clinically relevant. It has been identified in 46.2% of platinum-resistant ovarian carcinomas that developed PARPi resistance and in 66.7% of patients with BC with previous chemotherapy history who later progressed on PARPi therapy^7^. Therefore, elucidating the mechanisms of PARPi resistance and developing strategies to overcome them will be crucial for prolonging the PFS and overall survival (OS) in affected patients.

This review explores recent advancements in the antitumor mechanisms of PARPi, their clinical applications and the development of both primary and acquired resistance to PARPi in BC. In addition, it highlights potential strategies to overcome these challenges.

## The antitumor mechanism of PARPi

**Fig. 1:**
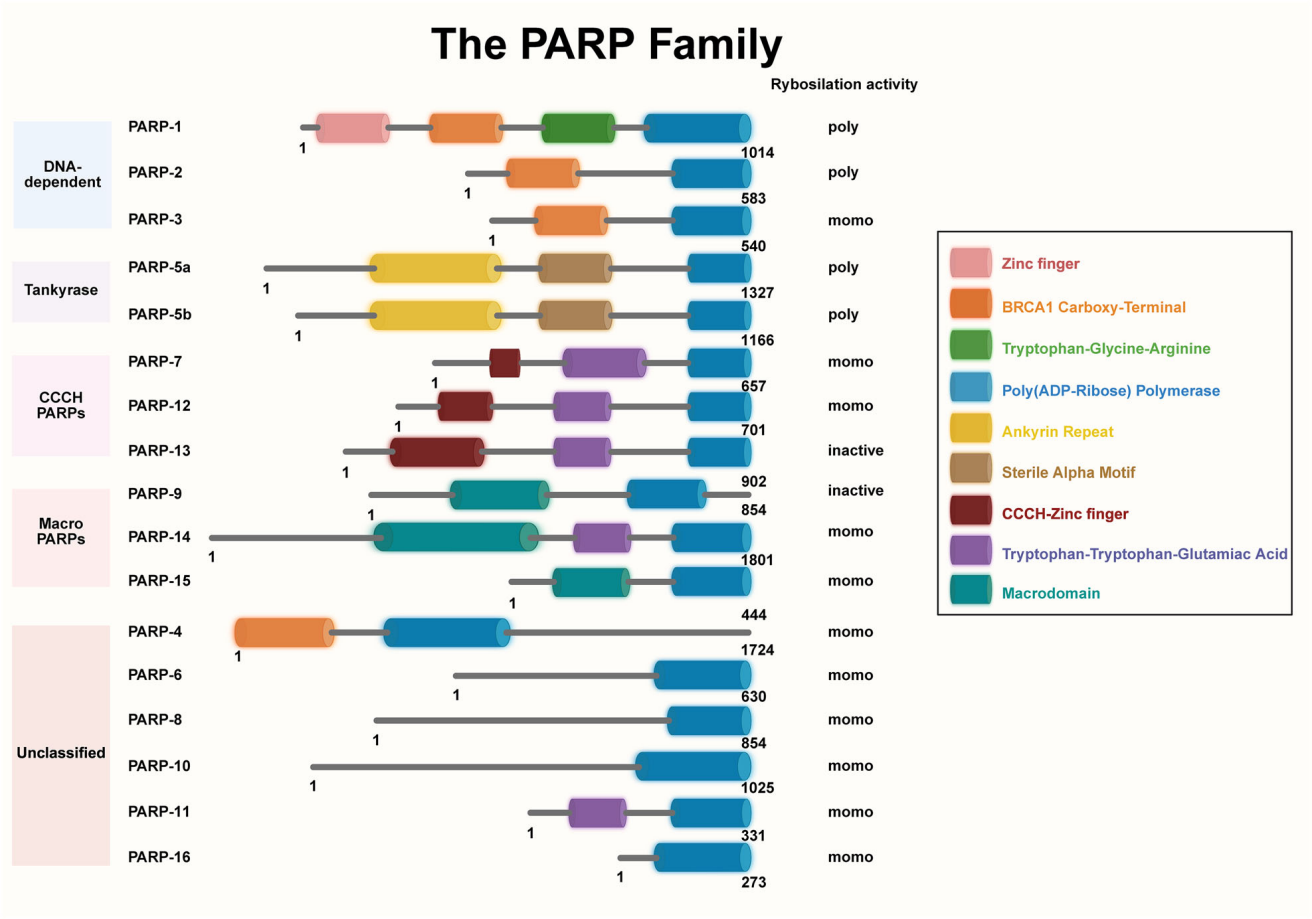
Structure of PARP family proteins. The schematic diagram depicts the domain organization of major PARP family members, categorized into DNA-dependent PARPs, Tankyrases, CCCH PARPs, Macro PARPs, and unclassified members. Key functional domains are annotated, including the poly(ADP-ribose) polymerase catalytic domain, zinc fingers, ankyrin repeats, and macrodomains. Numerical labels (e.g., PARP-1: 1014) indicate the total amino acid length of each protein. Figure 1 was created using Biorender (https://biorender.com/). Publication rights were acquired under a paid subscription in 2025.

**Fig. 2:**
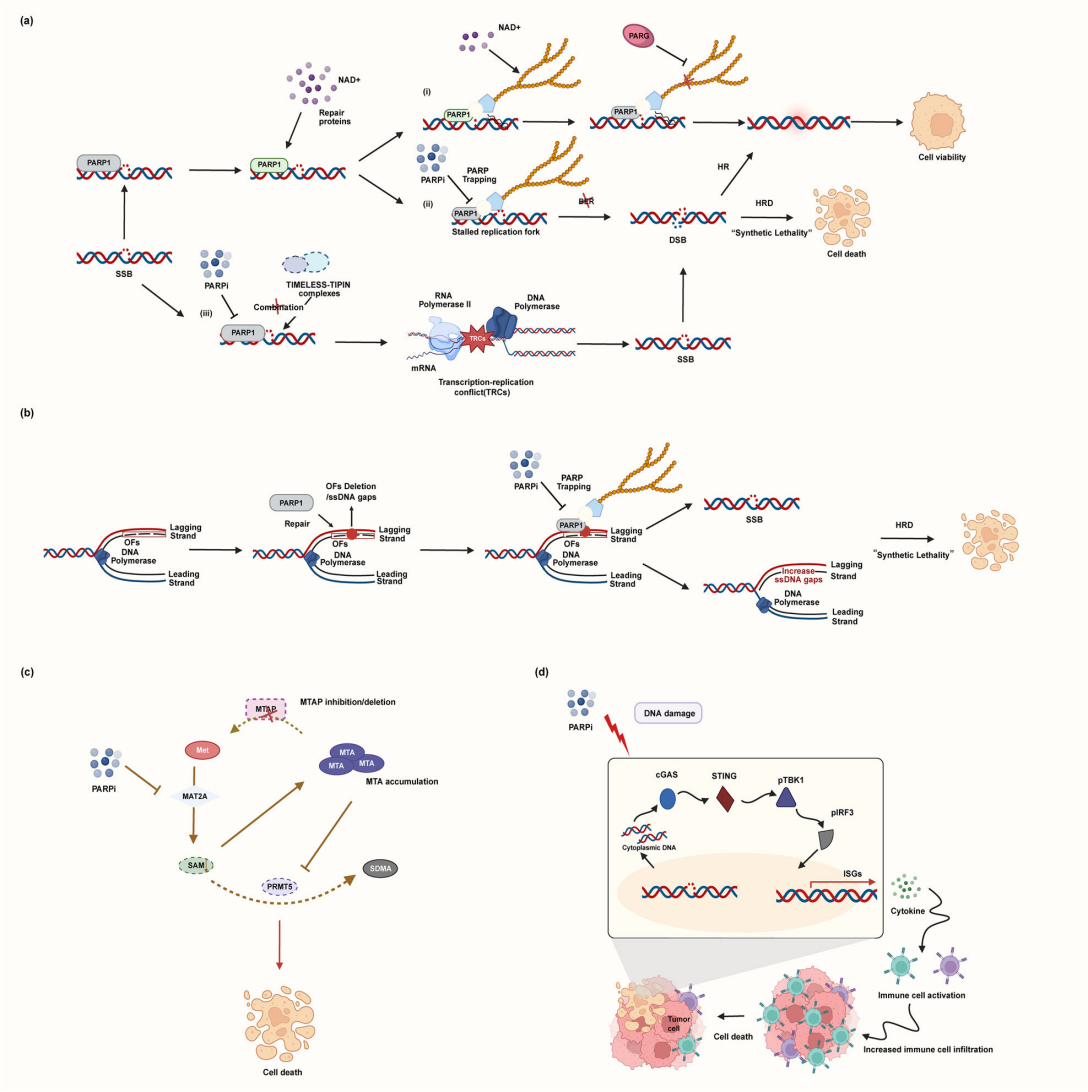
Antitumor mechanisms of PARPi. The diagram shows the diverse mechanisms through which PARPi exert their antitumor effects, extending beyond classical synthetic lethality. **a** PARPi and HR deficiency. (i) PARP1 detects DNA SSBs and catalyzes poly(ADP-ribosyl)ation to recruit repair proteins. Poly(ADP-ribose) glycohydrolase (PARG) hydrolyzes PAR chains to terminate the signal. (ii) PARPi traps PARP1 on DNA, preventing its release and disrupting repair. Unrepaired SSBs collapse into DSBs during replication. In HRD cells, these lethal DSBs cannot be faithfully repaired, leading to cell death via synthetic lethality. (iii) PARPi induce TRC and lethal cell death by inhibiting PARP1 activity. **b** PARPi and replication stress. PARPi traps PARP1 on DNA at damaged sites. This blockade prevents the normal progression of the replication fork, leading to the formation of ssDNA gaps and collapsed forks. **c** PARPi induce SAM reprogramming. PARPi inhibits the methionine cycle by downregulating MAT2A, reducing SAM synthesis. In cells with MTAP deficiency or inhibition, the metabolite MTA accumulates and further inhibits MAT2A, creating a feed-forward loop that depletes SAM, disrupts cellular methylation and synergizes with PARPi to induce cell death. **d** Immunostimulatory effects of PARPi. PARPi-induced DNA damage and replication stress lead to the accumulation of cytoplasmic DNA. This DNA is sensed by cGAS, which activates the STING–TBK1–IRF3 signaling axis, resulting in the production of interferons and other interferon-stimulated genes. This innate immune response promotes the activation, recruitment and infiltration of CD8^+^ T cells and dendritic cells, enhancing antitumor immunity and contributing to tumor cell killing. Figure 2 was created using Biorender (https://biorender.com/). Publication rights were acquired under a paid subscription in 2025.

PARPs are a family of nuclear enzymes, with around 17 known members (Fig. 1). These enzymes catalyze the addition of ADP-ribose units from nicotinamide adenine dinucleotide (NAD^+^) molecules to target proteins, resulting in either mono-ADP-ribosylation or poly-ADP-ribosylation^8^. Among these, PARP1 is the most well studied (Fig. 2a). The basal enzymatic activity of PARP1 is relatively low but increases markedly under DNA damage. The targets of PARP1 include PARP1 itself, histones and several DNA repair-related factors, which highlight the essential role of PARP1 in DNA repair processes.

Several PARPi have been developed and used in clinical applications. While the antitumor effects of PARPi through synthetic lethality in homologous recombination (HR) repair deficiency and replication fork destabilization have been well established, recent studies have further advanced our understanding of their therapeutic potential. Emerging evidence suggests that PARPi not only affect DNA repair but also play a role in reprogramming cancer cell metabolism and enhancing the efficacy of immunotherapy.

### Primary cytotoxic mechanisms of PARP trapping, catalytic inhibition and replication stress

PARPi mimic NAD^+^ and bind to the catalytic domain of PARP1, thereby blocking its enzymatic activity and trapping PARP1 on DNA. This inhibition leads to the accumulation of unrepaired single-strand breaks (SSBs) and, simultaneously, the formation of PARP–DNA complexes that obstruct replication forks. These obstacles cause fork collapse and the generation of lethal double-strand breaks (DSBs)^9^. Cancer cells with *BRCA* gene defects are unable to repair DSBs by HR, making them selectively sensitive to PARPi. This highlights a synthetic lethal relationship between HR deficiency and PARP trapping (Fig. 2a).

BRCA1 and BRCA2 play crucial roles in HR repair. A recent study demonstrated that PARPi treatment causes PARP1 retention at resected DNA in *BRCA2*-deficient cells, but not in *BRCA2* proficient cells, thereby disrupting RAD51-mediated DNA strand exchange^10^. In *BRCA2*-deficient tumors, PARPi treatment leads to the destabilization of RAD51 nucleofilaments, impairing HR repair. Cells deficient in *BRCA1*/*2* cannot effectively suppress the activation of replication origins or repair DSBs, ultimately causing cell death^11^. Notably, different PARPi exhibit varying capacities to disrupt RAD51 filament formation, with this ability correlating directly to their efficiency in inducing PARP1 retention at DNA lesions. Inhibitors with higher PARP1 trapping capacity showed increased cytotoxicity in monotherapy^12^. Talazoparib demonstrates exceptional PARP trapping capabilities, potentially explaining its enhanced efficacy as a monotherapy compared with other PARPi^13^. Olaparib and talazoparib are comparable in inhibiting PARP catalytic activity, but the PARP-trapping ability of talazoparib is about 100 times that of olaparib. This makes talazoparib the most cytotoxic, especially in *BRCA2*-deficient cells. Thus, on the basis of current evidence, talazoparib is considered one of the most potent PARPi in clinical use^14^.

However, an emerging study has questioned the importance of PARP trapping in inducing cell death in *BRCA1*/*2*-deficient cells^15^. PARP1, in conjunction with TIMELESS and TIPIN, can protect the replication fork from the effects of transcription–replication conflicts (TRCs). The DNA damage caused by TRCs requires HR for repair. The study found that the efficacy of PARPi inducing TRC-dependent DNA repair in HR deficient (HRD) cells during the S phase is related to the inhibition of PARP1 enzyme activity, rather than to the PARP trapping. Inhibiting the enzyme activity of PARP1 is sufficient to induce TRCs, thereby leading to DNA damage and cell death. Therefore, in *BRCA*-deficient cells, the inhibition of PARP1 activity exacerbates TRCs, leading to increased DNA damage that cannot be repaired through HR, which also induces synthetic lethality (Fig. 2a).

The relative contributions of PARP trapping versus catalytic inhibition to the overall cytotoxicity of PARPi remain a subject of ongoing scientific discussion. PARP trapping is widely regarded as a fundamental mechanism, strongly supported by the robust correlation between the trapping potency and the clinical efficacy of various PARPi^16^. However, emerging evidence suggests that the inhibition of the enzymatic activity of PARP1 is sufficient to induce lethal TRCs, independent of trapping. This notion is strongly supported by the development of a selective PARP1 degrader, 180055. Unlike traditional inhibitors that stabilize PARP1 on DNA, 180055 achieves potent antitumor efficacy by degrading PARP1 through the ubiquitin–proteasome system, thereby inhibiting its enzymatic function without causing substantial PARP1 trapping or exacerbating DNA damage^17^. This does not necessarily invalidate the trapping but rather highlights the complexity of the mechanism of action of PARPi. It is plausible that both mechanisms coexist and contribute to synthetic lethality, with their relative importance potentially varying on the basis of the cellular context, the specific PARPi used and the extent of the HR-deficiency. Despite this, the prevailing consensus, backed by substantial pharmacological and clinical evidence, continues to assign primary importance to PARP trapping as the dominant mechanism of action for the most effective PARPi. Future research aimed at dissecting the specific contexts in which each mechanism predominates will be crucial for fully optimizing PARPi-based therapies and managing resistance.

Beyond their influence in DSB repair, PARPi also profoundly affect DNA replication dynamics. Their impact is not limited to the direct obstruction of replication forks but includes more complex mechanisms. PARP inhibition impedes the maturation of the lagging strand during DNA replication, leading to an increase in postreplicative single-strand nicks or gaps (Fig. 2b). Numerous Okazaki fragments (OFs) are produced during the S phase, and the loss of any OF leads to elevated SSBs, resulting in cell cycle arrest, replication fork instability and increased genomic instability^18,19^. Defective OFs will activate PARP1 to repair SSBs and maintain the integrity of the newly synthesized DNA strand. PARP inhibition hinders the maturation of the lagging strand during DNA replication, resulting in an increase in SSBs or single-stranded (ss)DNA gaps after replication^20^. Some studies have shown that PARP inhibition does not obviously increase ssDNA gaps but rather causes ssDNA gaps to persist by trapping PARP1, which accumulates SSBs^21^. PARPi induces PARP1-trapped ssDNA gaps during the first S phase. If these gaps persist into mitosis and the subsequent S phase, they lead to the formation of DSBs^11^.

### PARPi reprograms SAM metabolism in cancer cells

*S*-adenosylmethionine (SAM) is a key methyl donor for protein and nucleic acid methylation in cells. SAM is synthesized by MAT2A, which uses methionine as a substrate^22^. In addition, the splicing of MAT2A pre-mRNA is tightly regulated by RNA methylation transferase METTL16, which responds to the availability of methionine in the environment^23^. Zeng et al. reported that PARPi activates ATM-mediated METTL16 phosphorylation, inducing a conformational change that disrupts the interaction between METTL16 and MAT2A pre-mRNA^24^ (Fig. 2c). This disruption inhibits MAT2A pre-mRNA splicing, leading to its degradation, ultimately reducing MAT2A expression and decreasing SAM synthesis.

MTAP plays a pivotal role in the methionine salvage pathway by converting methionine adenosylate to methionine^25^. MTAP deficiency creates a synthetic lethal effect when combined with inhibition of the METTL16–MAT2A axis. Cancer cells with MTAP deficiency or inhibition exhibit increased sensitivity to PARPi. Notably, because of the limited availability of methionine in the interstitial fluid or cerebrospinal fluid of the brain, TNBC with brain metastasis is more likely to benefit from PARPi treatment, particularly when MTAP is deficient or inhibited.

### PARPi boosts cancer immunotherapy

The introduction of immune checkpoint blockade has revolutionized oncology, facilitating the approval of several therapeutic agents across various cancer types. Notably, immune responses have been shown to play a role in the antitumor effects of PARPi monotherapy in TNBC. Constantia et al. demonstrated that olaparib induces infiltration and activation of CD8^+^ T cells within TNBC tumors^26^. This T cell recruitment is mediated through the activation of the cGAS–STING pathway in tumor cells. The response is notably more pronounced in HR-deficient TNBC cells compared with HR-proficient cells. Furthermore, knockout of STING or depletion of CD8^+^ T cells greatly diminishes the antitumor efficacy of olaparib. Preclinical models (patient-derived xenografts and mouse allografts) demonstrated that BCs with acquired PARPi resistance develop an immune-cold microenvironment with suppressed interferon signaling, which can be overcome by combining PARP inhibition with a STING agonist to reactivate the cGAS–STING pathway^27^. Consistent with this mechanism, a better PARPi response in *BRCA1*/*2* mutant tumors correlated with increased infiltration of CD56+ natural killer cells.

Current studies were focused on further improving the efficacy of immunotherapies and expanding their clinical applications. One promising strategy is combining PARPi with immune checkpoint inhibitors (ICIs). This synergy is rooted in a defined mechanism: PARPi-induced DSBs lead to the accumulation of cytosolic DNA, which activates the cGAS–STING pathway. This activation subsequently upregulates chemokines, enhancing antitumor immunity (Fig. 2d).

In addition to the critical roles of PARP1/2 in clinical applications, PARP7 has also emerged as a key player in modulating immune responses. PARP7 has been reported to inhibit IFN-β expression, contributing to the immunosuppressive environment. Given its role in suppressing IFN-β-mediated antitumor immunity, PARP7 inhibitors, such as RBN-2397, are gaining attention as potential agents to reverse immune evasion. Preclinical studies have shown that RBN-2397 not only restores IFN-β production but also synergizes with immune checkpoint blockade (for example, anti-PD-1) by enhancing CD8^+^ T cell infiltration^28^. Clinically, the PARP7 inhibitor RBN-2397 is currently undergoing phase I trials (NCT04053673), with preliminary data suggesting its potential to activate intratumoral T cells^29^. These findings establish a link between the molecular function of PARP7 and its immunomodulatory effects, providing a compelling rationale for combining PARP7 inhibitors with immunotherapy in cancer treatment.

## Clinical development and application of PARP inhibitors

The comprehensive elucidation of the antitumor mechanisms of PARPi, as detailed in the previous section, has provided a solid scientific foundation for their clinical development. This mechanistic understanding has directly fueled the translation of PARP-targeted therapy into clinical practice, leading to the successful development and regulatory approval of several potent PARP1/2 inhibitors. The following subsections review the key agents, their pivotal clinical trials and the evolving landscape of their application in BC.

The clinical development of PARPi began with early-phase trials combining rucaparib and temozolomide in advanced solid tumors. However, these initial studies failed to establish an optimal therapeutic window owing to dose-limiting toxicities^30^. Those pioneering works subsequently catalyzed the development of multiple PARPi, culminating in the FDA approval of four compounds—olaparib, talazoparib, rucaparib and niraparib—for HRD cancers^31^. Among these inhibitors, olaparib and talazoparib have emerged as the primary therapeutic options for *BRCA*-mutated BC. In addition, although some drugs, such as veliparib, fuzuloparib and pamiparib, have not yet received FDA approval, extensive ongoing clinical research demonstrates their potential role in treating *BRCA*-mutated BC.

Pivotal clinical trials (OlympiA, OlympiAD, PETREMAC, EMBRACA and BROCADE3) have systematically evaluated PARPi efficacy both as monotherapy and in combination with chemotherapy for frontline treatment (Table 1). These studies consistently demonstrate significant PFS benefits in HRD populations compared with standard therapies^32–35^. However, various PARPi may have different cytotoxic potential and toxic side effects on tumor cells owing to their different ability to induce PARP reverse conformation, which makes the OS outcome of patients with BC show considerable variability: whereas EMBRACA and BROCADE3 reported statistically significant OS improvements, OlympiAD revealed only a nonsignificant trend toward OS benefit.

**Table 1:** Summary of PARPi under clinical investigation for BC.

PARP inhibitor	Key trials	Target population	Treatment strategies	Key outcomes	Reference
**Olaparib**	OlympiA	HER2-negative early BC with g*BRCA1*/*2*m^1^	Olaparib + carboplatin/paclitaxel (O) versus placebo + carboplatin/paclitaxel (P)	O versus P in terms of the 3-year iDFS^2^ was 85.9% versus 77.1%	^3^
	OlympiAD	Patients with a g*BRCA1*/*2*m^1^ and HER2-negative mBC^3^	Olaparib (*n* = 205) versus TPC (capecitabine, vinorelbine or eribulin) (*n* = 97)	Olaparib versus TPC in terms of mPFS^4^ was 7.0 versus 4.2 months; RR^5^ was 59.9% versus 28.8%; higher AEs were 36.6% versus 50.5%	^33,37^
	PETREMAC	Patients with primary TNBC >2 cm	Olaparib for up to 10 weeks before chemotherapy	18 out of 32 patients obtained an ORR^6^ to olaparib (56.3%)	^34^
	TBCRC 048	mBC^3^ with measurable disease and germline mutations in non-*BRCA1*/*2* HR-related genes (cohort 1) or somatic mutations in these genes or *BRCA1*/*2* (cohort 2)	Olaparib 300 mg orally twice a day until progression	Cohort 1: ORR^6^, 33% Cohort 2: ORR^6^, 31%	^96^
**Rucaparib**	ROI, RUBY	g*BRCA*-mutated, HER2-negative mBC^3^	<30 mg with chemotherapy (tested)	ctDNA^7^ suppression; CBR^8^ 32%	^39,40^
**Niraparib**	NCT03329937	HER2-negative, g*BRCA*-mutated, locally advanced BC	Varies (two to six cycles)	pCR^9^: 40%; no new safety issues	^42^
**Talazoparib**	EMBRACA	HER2-negative advanced BC with g*BRCA1*/*2*m^1^	Talazoparib (T) (*n* = 205) versus PTC (capecitabine, eribulin, gemcitabine or vinorelbine) (*n* = 97)	T versus PTC in terms of mPFS^4^ was 24.3 versus 6.3 months; ORR^6^ was 63.2% versus 37.9%; CBR^8^ was 74.5% versus 46.4%	^97,98^
**Veliparib**	S1416	mBC^3^ with g*BRCA1*/*2*m^1^, *BRCA*-like and non-*BRCA*-like	Veliparib + cisplatin (VCis) (*n* = 162) versus placebo + cisplatin (PC) (*n* = 158)	VCis versus PC in terms of mPFS^4^ was 5.9 versus 4.0 months	^48^
	BrighTNess	Stage II–III TNBC	Veliparib + paclitaxel + carboplatin (VPC) (*n* = 316) versus paclitaxel alone (P) (*n* = 158).	pCR^9^ 53% in VPC versus 31% in P	^99^
	BROCADE3	*BRCA1*/*2*-mutated, HER2-negative advanced BC	Veliparib + carboplatin/paclitaxel (V) (*n* = 337) versus P (*n* = 158)	V versus P in terms of mOS^10^ was 32.4 versus 28.2 months	^77^
	NCT01149083	g*BRCA1*/*2*m^1^ associated with mBC^3^	Veliparib + carboplatin (VC) (*n* = 27)	The RR^5^ was 56%, three patients in CR^11^ beyond 3 years	^78^
**Fuzuloparib**	NCT04296370	HER2-negative, g*BRCA*-mutated mBC^3^	150 mg twice daily	Improved ORR^6^ and PFS^12^ (ongoing trial)	^52^
**Pamiparib**	NCT03575065	HER2-negative, *BRCA1*/*2*-mutated (TNBC^13^ and ER+/HER2−)	Not specified	ORR^6^: 61.9% (ER+/HER2−); >25% (TNBC); manageable AEs	^53,54^

*RR*, response rate; *CBR*, clinical benefit rate; *pCR*, pathologic complete response; *mOS*, median overall survival; *CR*, complete response; *ER*+/*HER2*−, ER-positive and HER2-negative.

### Olaparib

Olaparib became the first FDA-approved PARPi in 2014 for the treatment of advanced ovarian cancer with germline *BRCA* mutations (g*BRCA*m). It has since demonstrated significant improvement in PFS in patients with breast and ovarian cancer and a superior safety profile compared with other PARPi. As a highly selective dual inhibitor, olaparib demonstrates potent activity against both PARP1 and PARP2, with enzymatic half maximal inhibitory concentration values of approximately 5 nM for PARP1 and 1 nM for PARP2^32^. These advantages have established olaparib as the most widely used and extensively investigated PARPi in clinical practice.

The clinical success of olaparib was strongly supported by robust preclinical evidence, which highlighted its efficacy both as a monotherapy and in combination with chemotherapeutic agents across various HR-deficient BCs^33,34^. In clinical trials, the dosage of olaparib was dependent on its formulation: the earlier capsule formulation was administered at 400 mg twice daily, while the later, more bioavailable tablet formulation is administered at 300 mg twice daily^32^.

The OlympiA trial, a randomized phase III study, enrolled 1,836 patients with high-risk, HER2-negative early-stage BC who had completed local treatment and (neo)adjuvant chemotherapy. It demonstrated that 1 year of adjuvant olaparib monotherapy significantly improved iDFS in patients with germline *BRCA1* or *BRCA2* mutations (85.9%) compared with placebo (77.1%)^3^. Separately, in the metastatic setting, the OlympiAD trial established the efficacy of olaparib in patients with germline *BRCA*-mutated, HER2-negative mBC, showing it extended median PFS (mPFS) compared with standard chemotherapy^36^. However, it is important to note that this PFS benefit did not translate into a statistically significant improvement in OS in the final analysis^37^. In addition, although generally manageable, hematologic toxicities such as anemia and neutropenia were more frequent in the olaparib group.

### Rucaparib

Rucaparib received initial FDA approval in 2016 for the treatment of *BRCA*-mutated ovarian cancer^38^. Although not currently approved for BC, its clinical activity has been explored in early-phase trials, such as the RUBY study, which reported limited efficacy. Investigations within these trials have established circulating tumor DNA (ctDNA) kinetics as a promising pharmacodynamic biomarker. The RIO clinical trial provided mechanistic evidence for the activity of rucaparib in BC, demonstrating statistically significant ctDNA suppression in patients with HR-deficient TNBC by the end of treatment^39^.

However, subsequent clinical evaluation in the phase II RUBY trial (NCT02505048) revealed limited efficacy. Rucaparib monotherapy achieved only a 32% clinical benefit rate in *BRCA*-mutated HER2-negative mBC (below the predefined efficacy threshold)^40^. Furthermore, concurrent administration of low-dose rucaparib (<30 mg in each cohort) with chemotherapy failed to improve PFS in *BRCA*-mutated TNBC, suggesting that this dosage does not provide sufficient PARP inhibition. While dose escalation could potentially enhance therapeutic efficacy, it is constrained by dose-limiting toxicities, particularly hematological AEs^41^.

These findings suggest that rucaparib may have limited clinical utility as a monotherapy for *BRCA*-mutated BC. Further investigation into optimized dosing strategies or rational combination approaches is needed to overcome these limitations.

### Niraparib

Niraparib, approved by the FDA in 2017, has demonstrated antitumor activity and a favorable safety profile in patients with locally advanced HER2-negative, *BRCA*-mutated BC. In a clinical trial, 40% of the patients achieved a pathological complete response (pCR) after receiving only niraparib (two to six cycles), with no new safety signals identified. However, the interpretation of pCR in this single-arm trial (NCT03329937) is limited by several factors: limited sample size, which reduces statistical power, therapeutic heterogeneity after neoadjuvant niraparib and variations in treatment durations (cycle numbers)^42^.

Despite these limitations, niraparib has shown strong efficacy in prolonging PFS in ovarian cancers with *BRCA* mutations and in those with high HRD scores. This suggests that addressing deficiencies may enable HRD-positive or *BRCA*-mutated BC to benefit from niraparib treatment^43^.

### Talazoparib

Following the elucidation of PARP trapping mechanisms, talazoparib received FDA approval in 2018. This agent combines potent PARP enzymatic inhibition combined with superior PARP trapping capacity, effectively suppressing tumor proliferation and inducing apoptosis^44^. The EMBRACA trial, a double-blind phase III study, randomized 431 patients with g*BRCA*m and HER2-negative, locally advanced BC or mBC to receive either talazoparib or physician’s choice of standard chemotherapy (TPC). Talazoparib was administered orally at a dose of 1 mg once daily in 21-day cycles. The results showed that talazoparib significantly improved mPFS by 3 months compared with the placebo group^45^.

Both the ABRAZO and EMBRACA trials indicated that while talazoparib treatment was associated with a high incidence of hematologic adverse events (AEs), only a minimal proportion of patients required permanent treatment discontinuation due to these AEs, as they were effectively managed through dose-adjustment strategies^46^.

### Veliparib

Veliparib, a PARPi in clinical trials, has relatively low PARP trapping activity due to its unique structure, which causes steric hindrance on binding to PARP1^47^. Unlike other PARPi, veliparib does not exhibit statistically significant antiproliferative activity as a monotherapy. The phase II S1416 trial in *BRCA*-mutated TNBC demonstrated that combining veliparib with cisplatin significantly prolonged mPFS to 5.9 months versus 4.2 months in the placebo group^48^.

Currently, the development of veliparib for BC is advancing through phase III clinical trials^49^. The BROCADE3 trial established that combining carboplatin–paclitaxel chemotherapy with veliparib during the treatment period, followed by veliparib maintenance therapy, significantly improved PFS in patients with germline *BRCA1*- or *BRCA2*-mutated HER2-negative advanced BC. The mPFS was 14.5 months in the veliparib combination group compared with 12.6 months in the placebo plus carboplatin–paclitaxel. This sequential treatment approach demonstrated durable clinical benefit, with the PFS advantage maintained at both 2- and 3-year follow-ups^35^.

### Fuzuloparib and pamiparib

Recently, China has approved two new PARPi, fuzuloparib and pamiparib, for the treatment of advanced ovarian cancer with g*BRCA*m in patients who have undergone two or more prior lines of chemotherapy^50^. This China-specific approval highlights the growing importance of regional development and evaluation of PARPi, addressing unmet medical needs in specific populations. Ongoing phase II/III clinical studies are evaluating the therapeutic potential of fuzuloparib in multiple solid tumor indications, including pancreatic, breast, prostate and lung cancers. In a phase I 3 + 3 dose-escalation trial, fuzuloparib demonstrated favorable pharmacokinetic profiling, with rapid absorption. At the recommended dose of 150 mg twice daily, the plasma concentration of fuzuloparib was maintained above 4 μg/ml, suggesting that the systemic concentration was sufficient to inhibit PARP activity without causing severe hematological toxicity^51^.

The NCT04296370 trial is evaluating fuzuloparib in patients with mBC with HER2-negative and g*BRCA*m, who had received two or more previous lines of chemotherapy, including anthracyclines and taxanes, and were either refractory or ineligible for endocrine therapy^52^. Although the trial is ongoing, fuzuloparib is expected to significantly increase PFS and objective response rate (ORR).

In a phase II clinical trial (NCT03575065), pamiparib was tested in patients with locally advanced or HER2-negative mBC harboring *BRCA1* or *BRCA2* mutations, who had received ≤2 previous lines of chemotherapy^53^. Participants received pamiparib monotherapy until disease progression or intolerable toxicity occurred. In the TNBC cohort, the ORR of pamiparib was significantly higher than the historical ORR of 25% observed with previous chemotherapy. In addition, patients who had not received platinum-based chemotherapy previously showed a higher response rate compared with those who had. In the ER+/HER2− cohort, pamiparib demonstrated a promising ORR of 61.9%, indicating strong antitumor activity.

The safety profile of pamiparib was consistent with that of other PARPi, with hematologic toxicities being the primary AEs. These could be effectively managed through dose adjustments and supportive care. The findings from these studies support pamiparib as a promising treatment option for patient with *BRCA* mutations^53,54^. Pamiparib’s approval by China’s National Medical Products Administration represents an important regional advancement, providing a critical therapeutic option for Chinese patients with advanced BC and underscoring the importance of local drug development strategies in addressing population-specific medical needs.

### Gaps in RWE

Although randomized controlled trials provide robust evidence for the efficacy and safety of PARPi, there is a growing recognition of the importance of real-world evidence (RWE) to complement these findings. RWE can offer insights into drug performance in broader, more heterogeneous patient populations, including those often underrepresented in clinical trials (for example, older patients, those with major comorbidities or those with diverse ethnic backgrounds)^55^.

For PARPi, key RWE gaps include: long-term safety and efficacy beyond clinical trials require further study, particularly in real-world settings. Key areas include management of hematologic toxicities, assessment of OS, optimal treatment sequencing, toxicity management and treatment adherence. Regional differences must also be examined, especially for agents such as fuzuloparib and pamiparib, which have distinct approval pathways and may be used in genetically diverse populations with different concomitant treatments. Addressing these gaps through well-designed prospective observational studies and analysis of large-scale, high-quality registries is crucial for optimizing the use of PARPi in clinical practice and informing health-care decisions.

## Mechanisms of PARPi resistance

**Fig. 3:**
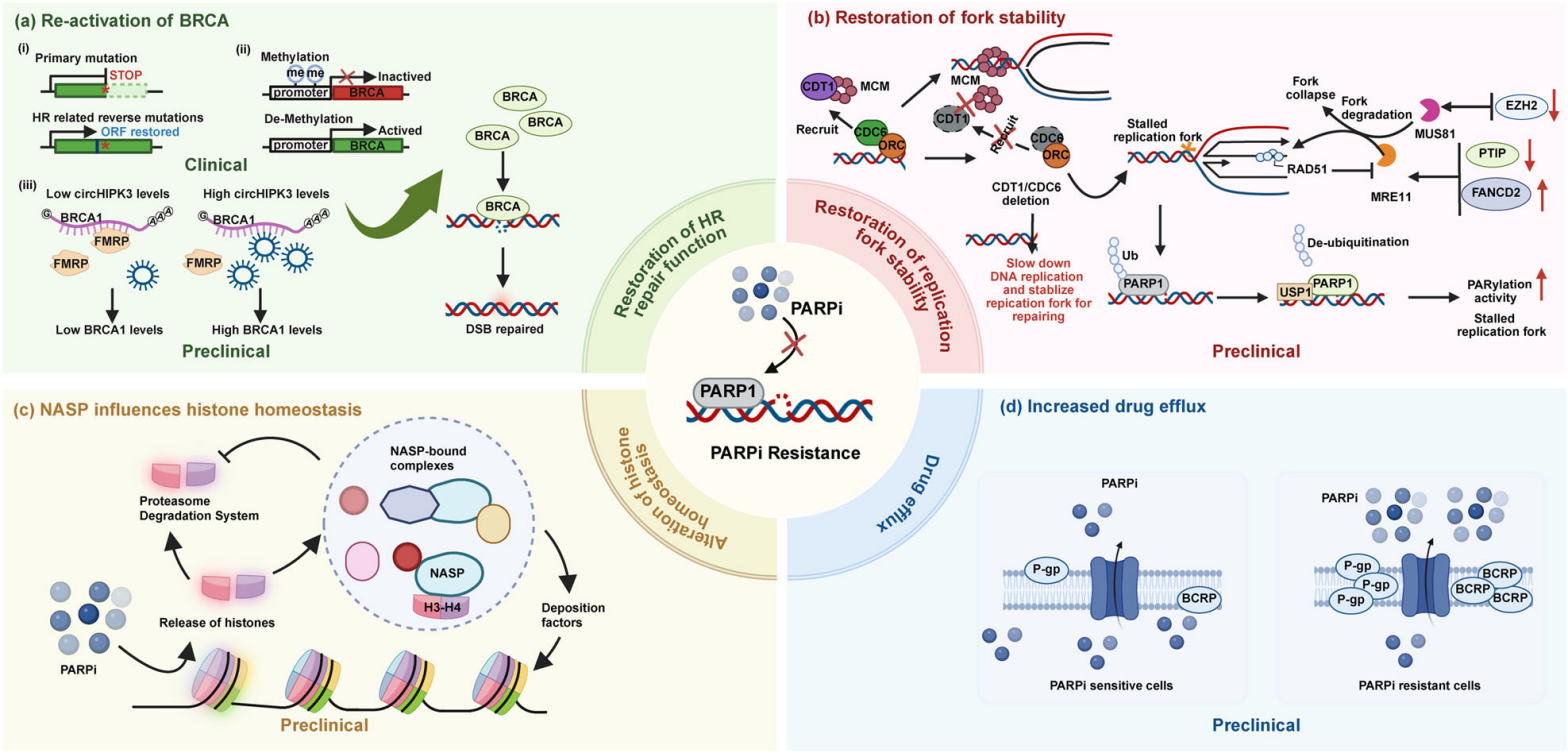
Mechanisms of PARPi resistance. The diagram shows four key molecular mechanisms through which cancer cells develop resistance to PARPi. **a** Reactivation of HR. (i) Reversion mutations: Secondary mutations in *BRCA1* or *BRCA2* genes can restore the ORF, leading to the production of functional BRCA proteins and restored HR. (ii) Promoter demethylation: Demethylation of the hypermethylated BRCA1 promoter can reactivate gene expression, restoring DNA repair capacity. (iii) CircHIPK3 regulation: The circular RNA circHIPK3 can bind to BRCA1 mRNA, counteracting the translational repression mediated by the RNA-binding protein FMRP, thereby increasing BRCA1 protein levels and promoting repair of DNA DSBs. **b** Restoration of replication fork stability. PARPi resistance is mediated by reduced recruitment of nucleases such as MRE11, MUS81 and PTIP to stalled replication forks, limiting fork degradation. In addition, loss of factors such as CDT1 or CDC6 decreases replication origin firing, thereby minimizing replication stress and fork collapse. EZH2-mediated silencing and deubiquitination of PARP1 by USP1 also contribute to fork protection. **c** Alteration of histone homeostasis. PARPi treatment induces histone release from chromatin. The histone chaperone NASP binds and stabilizes these histones (for example, H3–H4 tetramers), protecting them from proteasomal degradation. In the absence of NASP, histones are degraded, leading to histone depletion, genomic instability and increased PARPi sensitivity. **d** Increased drug efflux. Overexpression of drug efflux transporters, such as P-glycoprotein (P-gp/ABCB1), on the cell membrane actively pumps PARPi out of the cell. This reduces the intracellular concentration of the drug, diminishing its efficacy and leading to resistance. Figure 3 was created using Biorender (https://biorender.com/). Publication rights were acquired under a paid subscription in 2025.

PARPi has shown statistically significant efficacy in patients with breast or ovarian cancer with *BRCA* mutations. However, primary or acquired resistance develops in 40–70% of PARPi-treated patients, leading to either nonresponse or tumor relapse^56^. PARPi resistance primarily arises through several key mechanisms: restoration of HR repair capacity, stabilization of replication forks, alteration of histone homeostasis and elevated drug efflux (Fig. 3).

### Restoration of HR repair

The restoration of HR is a major clinical mechanism of PARPi resistance. Reversion mutations that restore the function of defective *BRCA1* or *BRCA2* genes are a key driver of this process. Clinical studies have revealed that these reversion mutations are detected in up to approximately 40% of patients with *BRCA*-mutated mBC who developed resistance to therapy^57^. A comparable mechanism is observed in ovarian cancer, where reversion mutations were identified in about 46% of platinum-resistant or PARPi-resistant hereditary carcinomas^7^. Prolonged PARPi exposure induces genomic instability, promoting reverse mutations that correct frameshift alterations or splice defects. These genetic events re-establish the open reading frame (ORF), re-express functional *BRCA1*/*2* and reactivate HR-mediated DNA repair, contributing to PARPi resistance^57^ (Fig. 3a). In a patient with ER+/HER2− BC who was resistant to olaparib, foundation one comprehensive genomic analysis was used to analyze the patient’s liver biopsy sample, and mutations in ESR1 Y537N and BRCA2 V1283fs*2 were detected. When the disease progressed after treatment with olaparib, a rearrangement mutation D1280_N1288del of *BRCA2* was detected by comprehensive genomic analysis. The D1280_N1288del is able to restore the ORF of *BRCA2*, thereby restoring the function of *BRCA2*^58^. The ARIEL2 phase II trial further revealed that reversion mutations in *RAD51C* or *RAD51D* can restore HR proficiency, mimicking the mechanism seen in *BRCA1*/*2*-reverted tumors, which leads to reduced sensitivity to rucaparib^59^.

In addition to genetic alterations, epigenetic dysregulation may also restore HR proficiency (Fig. 3a). Hypermethylation of the *BRCA1* promoter silences gene expression, sensitizing tumors to PARPi. Conversely, demethylation reactivates *BRCA1* transcription, reinstating DNA repair capacity and conferring resistance. Interestingly, even partial loss of methylation in one allele, among multiple BRCA1 copies, is sufficient to rescue HR defects and induce PARPi resistance^60,61^. This indicates that the methylation status (homozygous or heterozygous) of the *BRCA1* gene has a substantial impact on the therapeutic response to rucaparib. Rucaparib responses in BRCA1-methylated patient-derived xenograft models depended on homozygous (but not heterozygous) methylation. Resistance correlated with BRCA1 re-expression via promoter demethylation, consistent with clinical findings^62^. Circular RNA has been shown to play a role in regulating BRCA1 expression. Specifically, circHIPK3 binds to the final coding exon of BRCA1 mRNA and competes with the RNA-binding protein FMRP. This competition prevents FMRP from inhibiting BRCA1 translation, thereby increasing BRCA1 protein levels. In the absence of circHIPK3, BRCA1 expression decreases, leading to increased DNA damage and making cancer cells susceptible to DNA-damaging agents^63^.

### Restoration of replication fork stability

Replication fork stability serves as a critical determinant of PARPi sensitivity. Multiple molecular pathways converge to regulate fork protection in *BRCA1*/*2*-deficient cells. Genetic alterations affecting fork stabilization proteins can bypass PARPi-induced synthetic lethality, contributing to acquired resistance (Fig. 3b).

PTIP is a component of the MLL3/4 methyltransferase complex, with tandem BRCA1 C-terminal (BRCT) domains implicated in DNA damage response (DDR) and replication forks stability. Besides, PTIP mediates MRE11 recruitment to the site of damage to promote the degradation of stalled replication forks^64^. Therefore, in *BRCA*-mutated cells, the absence of PTIP leads to a reduction in the recruitment of MRE11 to stalled replication forks and a decrease in the degradation of newly synthesized DNA strands, thereby stabilizing the replication forks^65^. This results in a reduction in DSBs associated with replication forks collapse in *BRCA1*/*2*-deficient cells and confers PARPi resistance.

The Fanconi anemia (FA) pathway is involved in DNA repair. BRCA1/2 and some FA proteins (such as FANCD2) are located at the stalled replication forks, protecting the newly synthesized strand from excessive nucleolytic degradation. FANCD2 was shown to directly interact with the MRE11 and inhibits the activity of MRE11, which can protect stalled replication forks from degradation, thereby conferring PARPi resistance in *BRCA1/2*-deficient BC cell lines^66^.

The methyltransferase EZH2 can be localized at stalled replication forks where it methylates Lys27 on histone 3 (H3K27me3), mediating the recruitment of the MUS81 nuclease. Low levels of EZH2 reduce H3K27 methylation, prevent MUS81 recruitment at stalled forks and cause fork stabilization, which indicates that loss of function of the EZH2–MUS81 axis promotes PARPi resistance in *BRCA2*-deficient cells. Therefore, low expression levels of EZH2 or MUS81 can predict chemoresistance and poor prognosis in patients with *BRCA2*-mutated tumors^67^.

The overexpression of the deubiquitinating enzyme USP1 can promote the deubiquitination of PARP1, which enhances the activity of PARP1 and reduces the capture of PARP1 in chromatin, so that PARP1 can be released from the DNA damage site more quickly to repair the DNA damage and stabilize the replication cleavage leading to PARPi resistance^68^.

For patients with primary resistance, Kyrie et al. reported that deletions of genes associated with DNA prereplication complexes lead to PARPi resistance by stabilizing replication forks^69^. The deletion of genes such as *CDT1* or *CDC6* alters the process and regulation of DNA replication, stabilizing replication forks that should have been degraded, which enables *BRCA2*-deficient tumor cells to survive under replication stress induced by PARPi, resulting in resistance that is independent of *BRCA2* reversion mutation.

### Alteration of histone homeostasis

In addition to exploring the downstream effects of PARPi exposure to address resistance mechanisms, Sarah et al. recently identified that the histone chaperone NASP plays a key role in regulating PARPi resistance by influencing histone homeostasis^70^ (Fig. 3c). NASP helps to maintain the storage of both pre- and postnucleosomal H3–H4 histone tetramers, which supports continuous DNA replication. It is suggested that PARP1 may also assist in restoring chromatin structure through its chaperoning activity. PARPi treatment results in the release of histones from chromatin by the INO80 complex. Once released, H3–H4 tetramers are stabilized and protected from proteasomal degradation by the chaperone activity of NASP. In the absence of NASP, the released and newly synthesized histones are left unprotected and degraded. Consequently, NASP knockout cells experience histone depletion due to the proteasomal degradation of chromatin-released histones, resulting in genomic instability and increased sensitivity to PARPi.

### Drug efflux

Drug efflux refers to the process by which cells pump drugs from the inside to the outside of the cell through specific transport proteins, thereby reducing the drug concentration inside the cell and decreasing its effectiveness (Fig. 3d). Key drug efflux transporter proteins include P-glycoprotein (P-gp), multidrug resistance-associated proteins (MRPs) and BC resistance protein (BCRP). For instance, increased P-gp expression in tumor cells can effectively pump PARPi out of the cell, reducing the drug’s inhibitory effect on DNA repair and conferring resistance to PARPi^71^. Similarly, overexpression of the *ABCB1* gene, which encodes multidrug resistance protein 1 (MDR1), can also contribute to PARPi resistance^72^. In ovarian cancers and BCs with multiple *ABCB1* fusion events, inhibition of MDR1 has been shown to restore sensitivity to PARPi^73^.

Despite drug efflux being a well-known mechanism of resistance across various drug classes, the role of P-gp efflux pumps in PARPi resistance currently lacks robust clinical evidence. Therefore, future clinical research should focus on elucidating the clinical relevance of drug efflux mechanisms in PARPi resistance, which may lead to the identification of new therapeutic targets to overcome this form of resistance.

## Emerging strategies to overcome PARPi resistance

Although PARPi hold substantial promise for treating BC, their clinical effectiveness is commonly limited by primary and acquired resistance. Consequently, research has focused on overcoming this through combination therapies, integrating PARPi with modalities such as chemotherapy, ICIs and inhibitors of the ATR–CHK1–WEE1 and PI3K–AKT pathways^74,75^ .

### Chemotherapy and PARPi combination

Chemotherapy remains a cornerstone treatment for BC, particularly for patients with TNBC. Combining chemotherapy with PARPi can increase the DNA damage burden and overcome resistance to PARPi. The BROCADE II/III trials have confirmed that the combination of veliparib and chemotherapy can improve the prognosis of patients with mBC harboring *BRCA1*/*2* mutations without significantly increasing toxicity^76,77^. Another phase I/II trial demonstrated that veliparib, when combined with carboplatin, is both safe and effective for treating *BRCA*-mutated patients, showing superior efficacy in early combination therapy compared with monotherapy^78^. In addition, a meta-analysis indicated that PARPi combined with chemotherapeutic agents may offer viable options for germline *BRCA*-mutated, HER2-negative BC, including both TNBC and hormone receptor-positive subtypes^79^.

### ICI and PARPi combination

ICIs block tumor growth by disrupting immune evasion pathways. Programmed death ligand 1 (PD-L1) is a protein that is expressed on the surface of many tumor cells and binds to programmed death protein 1 (PD-1) on the surface of T cells and inactivates T cells, allowing tumor cells to evade immune surveillance^80^. Moreover, upregulation of PD-L1 promotes DSB repair, thereby inducing PARPi resistance, whereas PD-L1 inhibition may restore cellular sensitivity to PARPi^81^. A single-arm meta-analysis supports the favorable efficacy and safety of combining ICIs with PARPi in patients with advanced or metastatic TNBC^82^. Furthermore, PARPi induces profound DNA damage, leading to cytoplasmic DNA accumulation and subsequent activation of the cGAS–STING signaling pathway. This cascade triggers IFN-γ production, which upregulates PD-L1 expression on tumor cells via the JAK1–STAT1 signaling axis. Therapeutically, ICIs can exploit this immunogenic vulnerability and demonstrate synergistic efficacy when combined with PARPi in patients with *BRCA* mutations and BC^83^. Clinical trials investigating PARPi and ICI combinations are currently underway across various solid tumors, with sequential administration strategies emerging as an innovative approach to optimize therapeutic efficacy^84^.

Despite the compelling preclinical rationale, the clinical translation of PARPi and ICI combinations has encountered major challenges. Several pivotal trials in broader TNBC populations have yielded disappointing results, highlighting the gap between mechanistic synergy and clinical effectiveness in unselected patients.

The phase II trial (NCT02657889) evaluated the combination of niraparib and pembrolizumab in patients with metastatic TNBC. While the combination showed a signal of activity in the subset of patients with *BRCA* mutations, the ORR in the overall cohort was only 21%^85^. Although the JAVELIN PARP Medley trial demonstrated notable efficacy in biomarker-selected cohorts such as *BRCA*-altered ovarian cancer (ORR 63.6%), responses were more modest in other populations, including an 18.2% ORR in an unselected TNBC cohort^86^. These results underscore that the clinical benefit of PARPi and ICI combinations is not universal and appears largely confined to specific molecular subtypes, highlighting the critical need for predictive biomarkers and validation in randomized trials.

### ATR–CHK1–WEE1 inhibitor and PARPi combination

The ATR–CHK1–WEE1 pathway is crucial in the cellular response to DNA damage and replication stress. As a primary DNA damage sensor, ATR orchestrates the DNA damage response by activating CHK1. Activated CHK1 subsequently phosphorylates WEE1, triggering cell cycle arrest to facilitate DNA repair^87,88^. Inhibiting the ATR–CHK1–WEE1 pathway can make tumor cells more susceptible to PARPi by destabilizing replication forks. While the direct role of CHK1 and WEE1 in stabilizing replication forks remains uncertain, preclinical studies suggest that combining PARPi with WEE1 inhibitors effectively suppresses tumor growth. This combination also induces sustained effects even after discontinuing the drugs.

However, the clinical translation of these combinations is seriously challenged by dose-limiting toxicities, most notably myelosuppression. The synergistic disruption of DNA repair in both tumor and highly proliferative bone marrow cells can lead to severe neutropenia and thrombocytopenia, narrowing the therapeutic window^89^. To mitigate these toxicities, innovative scheduling strategies are being explored. Preclinical evidence suggests that sequential administration (initiating WEE1 inhibition before PARPi) may reduce damage to normal cells while maintaining antitumor efficacy^90^. Beyond sequential administration, a critical strategy to improve the therapeutic index of PARPi combinations lies in biomarker-driven patient selection. The key is to move beyond predicting sensitivity to PARPi alone and instead identify the specific molecular context that makes a tumor vulnerable to a synergistic interaction. For instance, in ovarian cancers exhibiting PARPi resistance and *CCNE1* amplification, replication stress driven by CCNE1 creates a critical dependency on the ATR–CHK1 pathway. Combining a CHK1 inhibitor in this setting further destabilizes replication forks, ultimately inducing lethal DNA damage and synergistically enhancing PARPi cytotoxicity^91^. This mechanism not only provides a novel combination strategy to overcome PARPi resistance but also establishes *CCNE1* amplification as a potential biomarker for predicting the efficacy of CHK1 inhibitors.

### PI3K/AKT inhibitor and PARPi combination

Inhibiting the PI3K–AKT pathway has been shown to increase DNA damage, reduce BRCA1/2 expression and enhance sensitivity to PARPi, particularly in TNBC^92^. The combination of PI3K inhibitors (for example, BKM120) with PARPi (for example, olaparib) induces DNA damage, thereby inhibiting tumor growth^93^. A preclinical trial evaluating the combination of talazoparib and LY294002 (a PI3K inhibitor) in patients with TNBC has demonstrated that the combination therapy is well tolerated and shows durable activity in women’s cancers^94^.

## Conclusions and perspective

PARPi has demonstrated significant clinical efficacy in treating patients with cancer with *BRCA1*/*2* mutations and other HR-deficient malignancies. However, several challenges persist. One key limitation is the relatively low frequency of HR-related gene mutations in BC, which restricts the broader applicability of PARPi in this population. In addition, the issue of hematologic toxicity and drug resistance further complicates the use of PARPi in clinical settings. These factors hinder the development of more targeted and effective PARPi treatment strategies.

To address these challenges, future research should prioritize several key areas:

### Developing novel inhibitors

One challenge with current PARPi is their lack of selectivity, as they broadly inhibit both PARP1 and PARP2, leading to substantial hematologic toxicity in clinical applications. Therefore, next-generation PARPi should aim to enhance drug selectivity and minimize off-target effects, thus broadening the therapeutic window while reducing toxicity. This strategy encompasses two key approaches: first, developing inhibitors with ultrahigh selectivity for PARP1 to mitigate hematologic side effects associated with PARP2 inhibition; and second, exploring inhibitors that target other members of the PARP family with specific biological functions. For instance, as mentioned earlier, the PARP7 inhibitor RBN-2397 can reverse tumor immune suppression by restoring type I interferon production and exhibits synergistic effects with anti-PD-1 therapy. RBN-2397 is currently in phase I clinical trials (NCT04053673), with preliminary data indicating its ability to effectively activate intratumoral T cells^29^. Similarly, inhibitors targeting PARP14, such as RBN012759, have shown potential in modulating the tumor immune microenvironment in preclinical models^95^. These highly selective inhibitors targeting specific PARP family members not only provide new avenues to overcome resistance to existing PARP1/2 inhibitors but also open up new frontiers in cancer immunotherapy.

### Optimizing combination therapies

The combination of PARPi with chemotherapy and ICIs shows promise, but issues such as chemotherapy-induced toxicity and individual variations in ICI responses need to be resolved. Further clinical trials are essential to determine the optimal combination strategies.

### Exploring resistance mechanisms and biomarkers

Resistance to PARPi remains a major challenge. Understanding the molecular mechanisms behind resistance and identifying biomarkers to predict PARPi response are crucial steps in advancing precision medicine. Integrating genomics, transcriptomics and proteomics analyses will permit the development of more effective and personalized treatment strategies, ultimately improving patient outcomes.

### Expanding focus beyond *BRCA* genes

While current studies predominantly focus on *BRCA* mutations, there is a need to shift greater attention to mutations in other HR-related genes. This broader focus will contribute to a deeper understanding of resistance mechanisms and potentially uncover new therapeutic targets.

### Translating basic research to clinical practice

Despite the many challenges, translating basic research findings into clinical practice is essential. When recruiting patients for clinical trials, researchers must consider the differences in molecular subtypes of BC and the associated resistance mechanisms. Investigating how PARPi interacts with various molecular subtypes and integrating PARPi into first-line treatment strategies for these subtypes may help overcome resistance and improve treatment outcomes.

### Addressing socioeconomic barriers to global access

Beyond the scientific and clinical challenges, the successful translation of PARPi therapy must also confront major socioeconomic barriers, particularly in low- and middle-income countries. The high costs associated with combination regimens (for example, PARPi with chemotherapy or ICIs), coupled with the requisite infrastructure for genetic testing and patient monitoring, creates substantial disparities in access. Ensuring equitable access to these therapies is a critical step toward reducing the global burden of BC.
